# Expression of Beclin-1 in the Microenvironment of Invasive Ductal Carcinoma of the Breast: Correlation with Prognosis and the Cancer-Stromal Interaction

**DOI:** 10.1371/journal.pone.0125762

**Published:** 2015-05-08

**Authors:** Akemi Morikawa, Tamotsu Takeuchi, Yusuke Kito, Chiemi Saigo, Takuji Sakuratani, Manabu Futamura, Kazuhiro Yoshida

**Affiliations:** 1 Department of Surgical Oncology, Gifu University Graduate School of Medicine, Gifu, Japan; 2 Department of Frontier Science for Surgical Oncology, Gifu University Graduate School of Medicine, Gifu, Japan; 3 Department of Pathology and Translational Research, Gifu University Graduate School of Medicine, Gifu, Japan; 4 Department of Breast and Molecular Oncology, Gifu University Graduate School of Medicine, Gifu, Japan; University of Alabama at Birmingham, UNITED STATES

## Abstract

We examined the pathobiological properties of beclin-1, which is a key regulator of autophagosome formation in invasive ductal carcinoma of the breast, with a particular focus on the cancer microenvironment. Immunohistochemistry demonstrated that cancer cells and stromal mesenchymal cells expressed beclin-1 in 68 and 38 of 115 invasive ductal cancers, respectively. Expression of beclin-1 in cancer or stromal cells alone did not correlate with patient prognosis. In contrast, loss of beclin-1 in cancer cells and overexpression in stromal mesenchymal cells was associated with local cancer recurrence, postoperative lymph node metastasis, and a poor disease-free survival rate. A comprehensive gene expression analysis was performed on a co-culture of breast cancer cells and mesenchymal stromal cells, that latter of which either expressed beclin-1 or was depleted of beclin-1 by siRNA. Notably, siRNA-mediated downregulation of beclin-1 in mesenchymal cells co-cultured with breast cancer cells decreased the levels of various pro-inflammatory cytokines, their receptors, and collagen receptors. Quantitative reverse transcription polymerase chain reaction analysis confirmed that reduction of stromal beclin-1 expression decreased the expression of *IL-1β* and collagen receptor *discoidin domain receptor 2 (DDR2)*. Microenvironmental IL-1β is believed to play an important role in tumor invasion. Recent work has also indicated that overexpression of DDR2 contributes to breast cancer invasion and lymph node metastasis. Taken together, these findings indicate beclin-1 expression in the stroma might be important for shaping the breast cancer microenvironment and thus could be a potent molecular target in patients with invasive ductal carcinoma of the breast.

## Introduction

Autophagy is the process of self-digestion in which lysosomal degradation is used to maintain cellular viability during periods of metabolic stress such as starvation [1,2]. During carcinogenesis, autophagy is important for tumor cell survival under starvation conditions. Paradoxically, autophagy defects are also associated with increasing carcinogenesis [3,4]. Beclin-1 is important at major step in autophagic pathways, from autophagosome formation, to autophagosome/endosome maturation. Beclin-1 is important at the major step in autophagic pathways, from autophagosome formation to autophagosome/endosome maturation [5,6]. Thus, beclin-1 is a key protein and reliable biomarker of autophagy in various cancers. Notably, although beclin-1 is normally expressed in the ductal epithelial cells of mammary glands, many breast cancer cells lack beclin-1 expression due to a combination of monoallelic deletion and epigenetic silencing of the *beclin-1* gene [5, 7]. Exogenous expression of beclin-1 in MCF-7 breast cancer cells promoted autophagy, inhibited cell growth, and decreased tumorigenesis in nude mice [5]. However, beclin-1-dependent autophagy is required for the tumorigenicity of breast cancer stem-like/progenitor cells [8]. Therefore, beclin-1 expression may have a dual role in breast carcinogenesis, acting both to promote and to suppress tumor progression.

Expression of beclin-1 in the tumor stroma also has an important role in the development of various cancers. The stromal cells play a crucial role in the progression of invasive breast cancer [9]. Autophagy in the cancer-associated stroma supports cancer progression metabolically through increasing glycolysis and ketone production in the tumor microenvironment [10]. Moreover, stromal autophagy plays a critical role in maintaining the tumor microenvironment to facilitate the growth of neighboring cancer cells [11]. Despite these findings, which highlight the important role of beclin-1 expression in the cancer-stromal niche, the combined effects of beclin-1 expression in tumor and stromal cells on prognosis of breast cancer patients remain unclear.

In this study, we first asked whether beclin-1 expression in invasive ductal carcinoma cells and/or stromal cells correlated with the prognosis of breast cancer patients. We subsequently examined the molecular background, which might reflect the clinicopathological findings, using a co-culture system comprising breast cancer- and bone marrow-derived mesenchymal cells.

## Materials and Methods

### Ethical statements

The paraffin-embedded tissues surgically resected from the patients were used as a retrospective study after its use for diagnosis. The need for written informed consent was waived by the Institutional Review Board of the Gifu University Graduate School of Medicine. Instead, the Institutional Review Board requested us to inform the patients that they could refuse to use their tissue specimens for this study, if they did not want to participate in the present study. The present study was conducted in accordance with the ethical standards of the Helsinki Declaration in 1975, after approval of the Institutional Review Board of the Gifu University Graduate School of Medicine (a specific approval number 25–81).

### Antibodies and immunohistochemical staining

A mouse-specific monoclonal antibody (clone 4H10) and a conventional rabbit antibody to beclin-1 were purchased from Novus Biologicals (Littleton, CO, USA) and Genetex (San Antonio, TX, USA), respectively. A normal rabbit antibody was also prepared in our laboratory.

Archived pathological tissue specimens from 115 invasive ductal carcinomas were used in this study. All tissue specimens were obtained surgically, fixed in 10% buffered formalin, and embedded in paraffin. Tissues were immunostained with antibodies using the ImmPRESS polymerized reporter enzyme staining system (Vector laboratories, Inc., Burlingame, CA, USA) as previously reported [12]. The tissue specimens were considered positive if the cancer cells or mesenchymal stromal cells exhibited more than 5% staining.

### Statistical analysis

Curves for disease free survival were drawn using the Kaplan-Meier method and the differences in survival rates were compared using the log-rank test for univariate survival analysis. A p value of <0.05 was considered statistically significant.

### Cell culture and siRNA-mediated RNA interference

UE6E7T-2 human bone marrow-derived mesenchymal cells, which have been used as a model of cancer-associated stromal mesenchymal cells [13, 14], were obtained from the RIKEN Biosource Center (Tsukuba, Japan). The MCF-7 and MDA-MB-157 breast cancer cell lines [15, 16], which are known to express little or no beclin-1 [5], were obtained from the Japan Health Science Research Resources Bank (Osaka, Japan) and American Type Culture Collection (Rockville, MD, USA), respectively. UE6E7T-2, MCF-7, and MDA-MB-157 were passaged in our laboratory for no more than 6 months after resuscitation.

The detailed procedure for siRNA silencing of a target gene has been described previously [12]. In this study, we employed the beclin-1 siRNA: 5’-CAGUUACAGAUGGAGCUAAtt-3’, which is located downstream from the initiation codon of the human beclin-1 coding region and has been used in previous reports.

We also used two other siRNA sequences, 5′-CUCAGGAGAGGAGCCAUUUtt-3′ and 5′-GAUUGAAGACACAGGAGGCUU-3′, for silencing the beclin-1 gene and then examined the expressions of *IL-1β*, *DDR2*, and *IL-10RB*. A green fluorescent protein (GFP) siRNA duplex with the target sequence 5’-CGGCAAGCUGACCCUGAAGUUCAU-3’ was used as a non-silencing control. siRNAs were transfected into UE6E7T-2 cells using lipofectamine RNAiMAX in accordance with the manufacturer’s instructions (Invitrogen, Carlsbad, CA, USA). At 48 h after transfection, the cells were used for subsequent studies.

Beclin-1 siRNA-treated or control UE6E7T-2 cells (1 × 10^4^) and MCF-7 or MDA-MB-157 cells (1 × 10^4^) were co-cultured in 24 well plates. After 48 h, total RNA was extracted from the co-cultured cells using RNeasy spin columns kit (Qiagen, Santa Clarita, CA, USA).

### Western blotting

Western blotting was performed according to the previously described method [17], in accordance with the proposal by Towbin et al. [18]. Cells were disrupted in Radio-Immunoprecipitation Assay (RIPA) buffer (Sigma-Aldrich, St. Louis, MO, USA), composed of 150 mM NaCl, 1.0% IGEPAL CA-630, 0.5% sodium deoxycholate, 0.1% SDS, and 50 mM Tris (pH 8.0) supplemented with a protease inhibitor cocktail (Nacalai Tesque, Kyoto, Japan), on ice for 15 min and then incubated at room temperature for 30 min with 0.25 U/μL of benzonase (Sigma-Aldrich). Then, these total cell lysates were mixed 1:1 with 2× SDS sample buffer (4% SDS, 20% glycerol, 0.12 M Tris (pH 6.8), 0.2 M DTT, and 0.02% bromophenol blue), incubated for 3 min at 95°C, and subjected to SDS-PAGE. The separated proteins were transferred onto polyvinylidene difluoride membranes (Millipore Co., Bedford, MA, USA) and probed with an anti-beclin-1 antibody (clone 4H10), anti-IL-1β antibody (Proteintech, Chicago, IL, USA), anti-actin antibody (Sigma-Aldrich), or anti-GAPDH antibody (Sigma-Aldrich). Immunoreactivity was assessed using a western blotting detection kit (Promega, Madison, WI, USA).

### cDNA microarray assay, reverse transcription polymerase chain reaction, and quantitative real-time reverse transcription polymerase chain reaction

We utilized the Human Whole Genome DNA Microarray system (SurePrint G3 Human 8x60K ver. 2.0, Agilent Technologies, Santa Clara, CA) to obtain the altered gene expression profile for co-cultured MCF-7 and UE6E7T-2 cells in which the *beclin-1* gene was silenced by siRNA. cDNA synthesis from the total RNA and subsequent PCR were performed using a reverse transcription polymerase chain reaction (RT-PCR) kit (Takara, Ohtsu, Japan). The procedure was performed according to the manufacturer’s instructions, as previously described [17].

The microarray data has been deposited to the GEO database with accession number GSE66154.

The Agilent-039494 SurePrint G3 Human GE v2 8x60K Microarray 039381 platform was used (Agilent, Santa Clara, CA, USA). Scanning and image analysis were performed using a DNA Microarray Scanner (Agilent). Raw fluorescence intensities were quantified and normalized using Agilent Feature Extraction Software (Agilent Feature Extraction 10.7.3.1). Normalization was performed with GeneSpring GX (Agilent). For processing, data were normalized by the percentile shift (all samples were normalized to the signal value corresponding to the 75th percentile of all of probes on the microarray).

To obtain the hierarchical combined tree for the 22 extracted genes reported to be related to inflammation or stromal reconstitution, we employed the GeneSpring Multi-Omic Analysis System (Agilent).

Real-time PCR reactions were performed using the SYBR Green reaction kit according to the manufacturer’s instructions (Roche Diagnostics, GmbH, Mannheim, Germany) in a LightCycler (Roche Diagnostics). cDNA (2 μL each) was diluted to a volume of 20 μL with the PCR mix containing a final primer concentration of 0.2 pmol.

The following primers were used for real-time RT-PCR: qPCR primers for human beclin-1-forward 5′-ACCGTGTCACCATCCAGGAA-3′, beclin-1-reverse 5′-GAAGCTGTTGGCACTTTCTGT-3’; GAPDH-forward 5′-GAAATCCCATCACCATCTTCCAGG-3′, GAPDH-reverse 5′-GAGCCCCAGCCTTCTCCATG-3′; IL-1β-forward 5′-AAAGAGGCACTGGCAGAA-3′, IL-1β-reverse 5′-AGCTCTGGCTTGTTCCTCAC-3′; DDR2-forward 5′-AGTCAGTGGTCAGAGTCCACAGC-3′, DDR2-reverse 5′-CAGGGCACCAGGCTCCATC-3′; and IL10RB (IL-10 receptor, subunit beta)-forward 5′-GGGGTCGTGTGCTTGGAG-3′, IL10RB-reverse 5′-GGTACCATTCCCAATGCTGA-3′.

To ensure that the SYBR green was not incorporated into primer dimers or non-specific amplicons during the real-time PCR runs, the PCR products were analyzed by polyacrylamide gel electrophoresis in preliminary experiments. Single bands of the expected sizes were obtained in all instances. The samples were cultured in triplicate, and the expression of each target gene was analyzed by the 2^-ΔΔCT^ method of Livak and Schmittgen [19] using the LightCycler system. The ΔC_T_ values were normalized to GAPDH for each triplicate set in both the si-GFP-treated (control) and si-beclin-1-treated groups. The values for the si-beclin-1-treated group were then calculated for each target gene as the fold change relative to the mean values for the si-GFP-treated group (control; set to 1.0). Then, the standard deviations were computed for the triplicate sets, namely, the 3 target genes and the fold changes are presented. In addition, Student’s t-tests were performed to determine significant differences. P < 0.05 was considered to be statistically significant.

## Results

### Expression of beclin-1 in invasive carcinoma tissue specimens

In this study, we predominantly used a murine monoclonal antibody to beclin-1 (clone 4H10 from Novus Biologicals) for immunohistochemical staining, but we also used another commercially available conventional rabbit antibody to beclin-1 on several tissue specimens to confirm the results. We obtained similar results with both antibodies. Significant immunoreactivity was not found using the control rabbit antibody.

**Fig 1:**
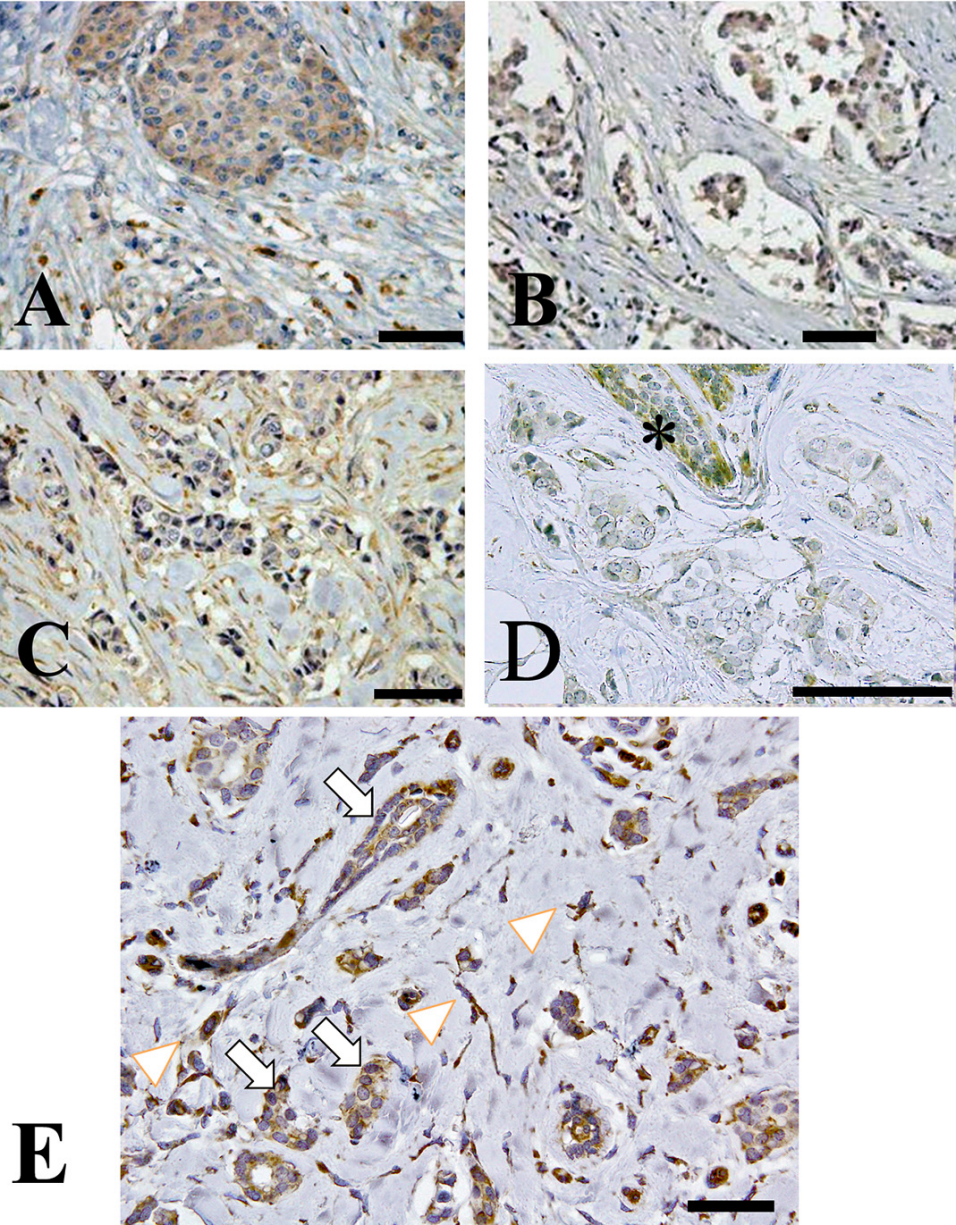
Representative immunohistochemical staining. (A) Both invasive ductal carcinoma and cancer stromal cells showing immunoreactivity with a specific monoclonal antibody to beclin-1. (B) Invasive ductal carcinoma cells, not the stromal cells, exhibiting beclin-1 immunoreactivity. (C) Significant immunoreactivity is not observed in the invasive ductal cancer cells, whereas the cancer-associated stromal cells exhibit beclin-1 immunoreactivity. (D) Neither invasive ductal cancer cells nor stromal cells showing beclin-1 immunoreactivity. Note the beclin-1 immunoreactivity in non-tumorous ductal epithelial cells (indicated by asterisk). (E) Cancer and stromal cells exhibiting beclin-1 immunoreactivity are indicated by arrows and arrow heads, respectively. Scales bars: 100μm.

**Fig 2:**
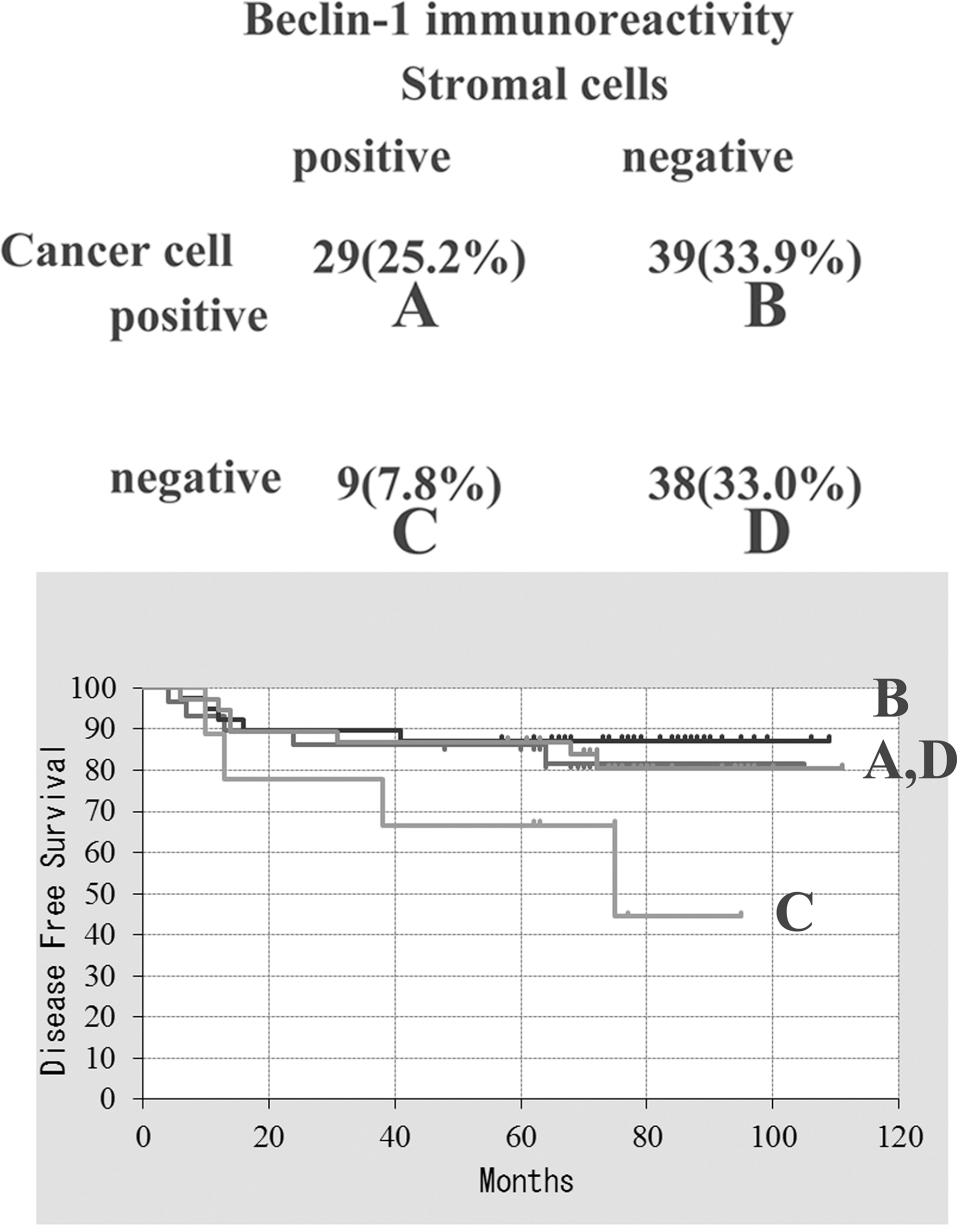
Disease-free survival curves according to beclin-1 expression in cancer and stromal cells. Lack of beclin-1 expression in cancer cells with concomitant stromal beclin-1 expression (indicated as C) was significantly associated with a poor disease-free survival rate (p = 0.0299<0.05).

Representative results from the immunohistochemical staining are shown in Fig 1. In this study, we examined beclin-1 expression in archived pathological tissue specimens from patients with invasive ductal carcinoma. Immunoreactivity of the anti-beclin-1 antibody was observed in the cancer cells of 68 of 115 tissue specimens and in the mesenchymal stromal cells of 38 of 115 invasive carcinoma tissue specimens (Fig 2). The absence of beclin-1 expression in the cancer cells alone was not significantly associated with a poor patient prognosis. Stromal beclin-1 expression was most often associated with a poor patient prognosis but this was not statistically significant. In contrast, lack of beclin-1 expression in cancer cells with concomitant stromal beclin-1 expression was linked to local cancer recurrence and postoperative lymph node metastasis, and thus was significantly associated with a poor disease-free survival rate (Fig 2).

### siRNA-mediated silencing of beclin-1 gene expression in mesenchymal stromal cells downregulated the expression of the *IL-1* agonist and *DDR2* genes, but upregulated the *IL-10RB* gene in a MCF-7 or MDA-MB-157 and mesenchymal stromal cell co-culture system

Next, we examined the molecular pathway responsible for the aggressive phenotype of beclin-1-negative cancer cells that have concomitant stromal beclin-1 expression. Our preliminary experiments demonstrated that MCF-7 and MDA-MB-157 breast cancer cells express low levels of beclin-1, as previously reported [5]

In contrast, several mesenchymal cell lines abundantly express beclin-1. In this study, we employed a bone marrow-derived, mesenchymal UE6E7T-2 cell line as a model of stromal cells for the following reasons. First, recent studies revealed that bone marrow-derived mesenchymal cells are important for the cancer-stromal microenvironment. Second, the usefulness of UE6E7T-2 mesenchymal cells and its series cells as a model of cancer stromal mesenchymal cells is well established [13, 14]. Third, preliminary experiments demonstrated that UE6E7T-2 cells allow regulation of beclin-1 expression by siRNA-mediated silencing, which was performed in this study.

**Fig 3:**
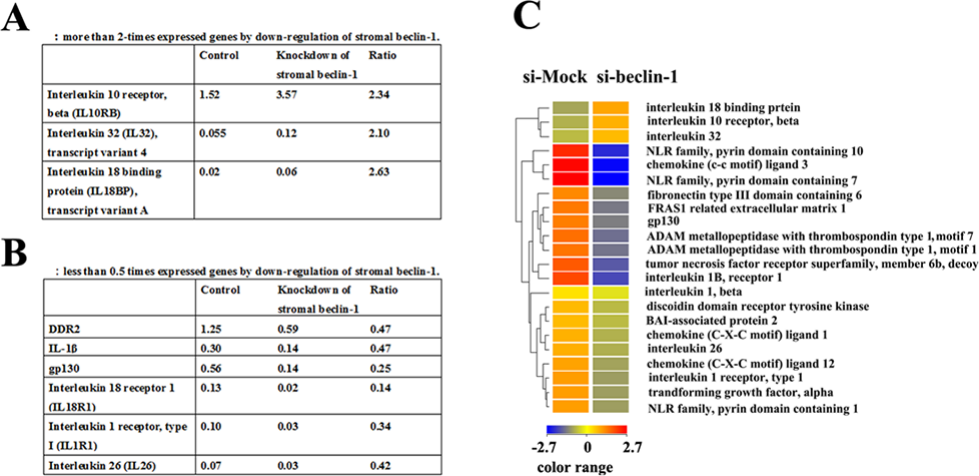
Summary of differentially expressed genes, as indicated by comprehensive microarray analysis, following downregulation of stromal beclin-1 expression, compared with the control MCF-7-UE6E7T-2 co-culture system. A: genes with greater than 2-fold expression after downregulation of stromal beclin-1. B: genes with less than 0.5-fold expression after downregulation of stromal beclin-1. C: hierarchical combined tree of the 22 extracted genes related to inflammation and stromal reconstitution in the cancer microenvironment.

Genes that showed a greater than 2-fold difference in expression (by cDNA microarray assay, S1 and S2 Datasets) between the co-cultures of MCF-7 cells with either beclin-1-expressing or beclin-1-downregulated UE6E7T-2 cells are summarized in Fig 3.

Notably, downregulation of beclin-1 in UE6E7T-2 cells decreased the expression of several pro-inflammatory cytokines and agonists such as *IL-1β*, *interleukin 18 receptor 1* (*IL18R1*), and *gp130*, but increased the expression of anti-inflammatory genes such as *interleukin-10 receptor beta* (*IL-10RB*) and *interleukin 18 binding protein* (*IL18BP*). Moreover, downregulation of beclin-1 decreased *DDR2* expression, which was recently shown to be critical for stabilizing SNAIL1 to promote breast cancer invasion and lymph node metastasis [20, 21]. In additional experiments, the viability of co-cultured cells exceeded 95% in both si-GFP- and si-beclin-1-treated groups. The ratios of the MCF-7 and UE6E7T-2 populations were preserved in si-GFP- and si-beclin-1-treated groups (S4 Fig).

**Fig 4:**
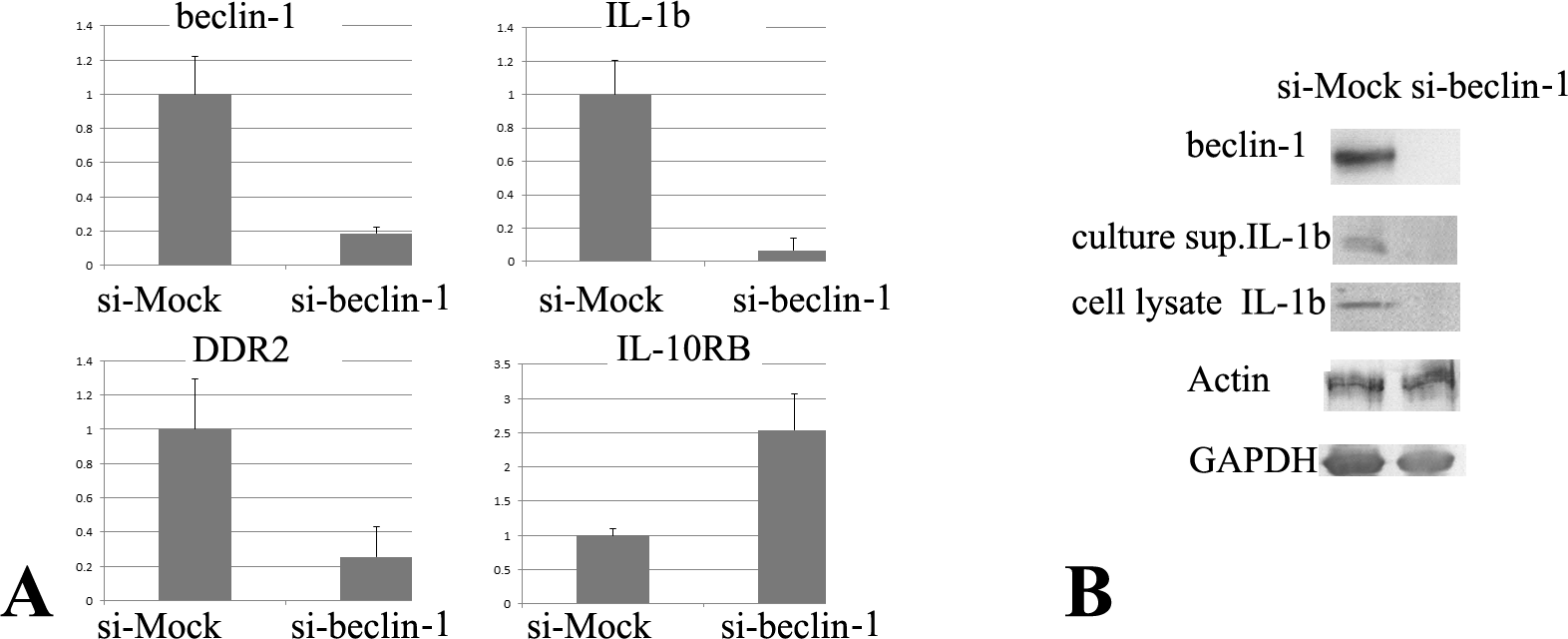
Representative quantitative reverse transcription polymerase chain reaction results and immunoblotting. siRNA treatment successfully reduced *beclin-1* mRNA (representative data of quantitative RT-PCR) and beclin-1 protein (representative data of Western-immunoblotting) in stromal UE6E7T-2 cells compared to control siRNA treated cells. (A) Downregulation of beclin-1 in UE6E7T-2 stromal cells significantly decreased the expression of the *IL-1β* and *DDR2* genes, whereas that of *IL-10RB* was increased in co-cultured MCF-7 and UE6E7T-2 cells. Gene expression changes were computed in triplicate reactions per sample using the 2^−ΔΔCt^ method. The data are represented as relative expression values compared with the mRNA expression in si-GFP treated cells after the normalization with GAPDH mRNA expression. Error bars indicate standard deviations. All values represent statistically significant fold changes (beclin-1: 0.00014, IL-1β: 0.00012, DDR2: 0.00011, and IL-10RB: 0.00016). We performed three independent, quantitative RT-PCR experiments using different MCF-7-UE6E7T-2 co-culture systems and obtained similar results. (B) Downregulation of beclin-1 in UE6E7T-2 stromal cells significantly decreased the expression of the IL-1β protein secretion (indicated as culture sup) and production (indicated as cell lysate) in co-cultured MCF-7 and UE6E7T-2 cells.

In summary, we confirmed the microarray data that were focused on the *IL-1β*, *IL-10RB*, and *DDR2* genes. RT-PCR analysis demonstrated that the downregulation of beclin-1 in UE6E7T-2 cells significantly abrogated the expressions of *IL-1β* and *DDR2*, but increased the expression of *IL-10RB* in co-cultured MCF-7 and UE6E7T-2 cells (Fig 4). We performed three independent, quantitative RT-PCR experiments using different MCF-7-UE6E7T-2 co-culture systems and obtained similar results. We also found that *IL-1β* and *DDR2* gene expressions were decreased, whereas *IL-10RB* gene expression was increased, by the downregulation of UE6E7T-2-beclin-1 in a co-culture system using MDA-MB-157 cells, similar to that found using MCF-7 cells (S1 Fig). We also employed two other siRNA sequences to exclude off-target effects and obtained similar results (representative data are shown in S2 and S3 Figs).

These results indicate that stromal beclin-1 expression may be important for reconstitution of the cancer microenvironment of the stroma thorough the proinflammatory IL-1β pathway and the collagen receptor DDR2.

## Discussion

Immunohistochemical staining revealed that a combination of loss of beclin-1 expression in cancer cells and overexpression of beclin-1 in cancer stromal mesenchymal cells was linked to local cancer recurrence and postoperative lymph node metastasis in invasive ductal carcinoma. There are several conflicting reports regarding the prognostic value of beclin-1 expression in breast cancer. Dong et al. determined that low expression of beclin-1 was associated with a worse 5-year overall survival rate in ER-positive and HER2-negative breast cancer [22]. He et al. reported that high expression of beclin-1 was associated with a favorable prognosis in breast cancer [23]. In contrast, Won et al. found no correlation between beclin-1 expression and the cumulative survival of patients with invasive breast cancer [24]. In the present study, beclin-1 expression in breast cancer cells alone was not significantly associated with patient prognosis, consistent with the data reported by Won et al [24]. We demonstrated that low expression of beclin-1 in breast cancer cells and high expression of beclin-1 in cancer stromal mesenchymal cells significantly correlates with a poor patient prognosis. Interestingly, lack of beclin-1 expression in cancer cells and overexpression of beclin-1 in stromal cells tends to be associated with local recurrence and postoperative lymph node metastasis.

Microarray analysis indicated that the downregulation of beclin-1 expression in bone marrow-derived mesenchymal UE6E7T-2 cells, which were co-cultured with MCF-7 cells, resulted in abrogation of the expression of several pro-inflammatory genes and *DDR2*, while upregulating *IL-10RB*, which is a receptor for the well-characterized anti-inflammatory cytokine IL-10. As shown in Fig 4, quantitative RT-PCR experiments confirmed that beclin-1 downregulation decreased *IL-1β* and *DDR2* expression, but increased *IL-10RB* expression. We speculate that beclin-1 expression in the stromal cells may be important to maintain the levels of IL-1 and to maintain mesenchymal collagen-binding receptors in the active state in cancer-stromal microenvironments. Importantly, Zhang et al. recently determined that DDR2 post-transcriptionally stabilizes SNAIL1 to promote breast cancer invasion and migration [20]. Moreover, DDR2 expression appears to be significantly associated with breast cancer lymph node metastasis [21]. It is likely that continuous DDR2 expression, which is related to stromal beclin-1 expression, plays a role in local recurrence and lymph node metastasis. In addition, a high IL-1β content within the breast cancer microenvironment is associated with tumor invasiveness [25]. Additional studies are in progress to unravel the molecular pathways by which beclin-1 expression or autophagy contribute to the breast cancer stromal microenvironment. In conclusion, the results from this study indicate that stromal beclin-1 expression could be a potent molecular target in the absence of beclin-1 expression in breast cancer cells.

## Supporting Information

S1 Fig Representative quantitative reverse transcription polymerase chain reaction results and immunoblotting using co-cultured MDA-MB-157 and UE6E7T-2 cells. (TIF)

S2 Fig Representative quantitative reverse transcription polymerase chain reaction results and immunoblotting using co-cultured MCF-7 and UE6E7T-2 cells with si-RNA, 5′-CUCAGGAGAGGAGCCAUUUtt-3′, to silence the beclin-1 gene. (TIF)

S3 Fig Representative quantitative reverse transcription polymerase chain reaction results and immunoblotting using co-cultured MCF-7 and UE6E7T-2 cells with si-RNA, 5′-GAUUGAAGACACAGGAGGCUU-3′, to silence the beclin-1 gene. (TIF)

S4 Fig Immunocytostaining demonstrated that MCF-7 cells exhibited immunoreactivity with AE1/AE3 (anti-multi-Cytokeratin antibody), but not with anti-vimentin antibody. By contrast, UE6E7T-2 cells were stained with anti-vimentin antibody but not with AE1/AE3 antibody (A). At 48h after co-culture, the cells were collected by centrifugation, resuspended in 0.1ml of PBS, dropped on the slide glass, and dried at room temperature. After methanol fixation, the cells were immunostained with antibodies using the ImmPRESS polymerized reporter enzyme staining system (Vector laboratories). We calculated the stained cell numbers in representative x200 power field. We did not observe significant differences in the proportion of AE1/AE3- or vimentin-positive cells between the si-GFP- and si-beclin-1-treated groups. Representative vimentin and AE1/AE3 staining is shown in Figure (B), and the mean and SD are also shown (C). The experiments were performed in triplicate and significance was evaluated using the Student’s t-test. Scales bars: 100μm
(TIF)

S1 Dataset The list of downregulated genes in a MCF-7 and mesenchymal stromal cell co-culture system by siRNA-mediated silencing of beclin-1 gene expression in mesenchymal stromal cells. (XLSX)

S2 Dataset The list of upregulated genes in a MCF-7 and mesenchymal stromal cell co-culture system by siRNA-mediated silencing of beclin-1 gene expression in mesenchymal stromal cells. (XLSX)
