# Supplementary figures and images for: Expression of Beclin-1 in the Microenvironment of Invasive Ductal Carcinoma of the Breast: Correlation with Prognosis and the Cancer-Stromal Interaction
Source: PLoS One. 2015 May 8;10(5):e0125762. doi: 10.1371/journal.pone.0125762 (PMC4425636; doi:10.1371/journal.pone.0125762)

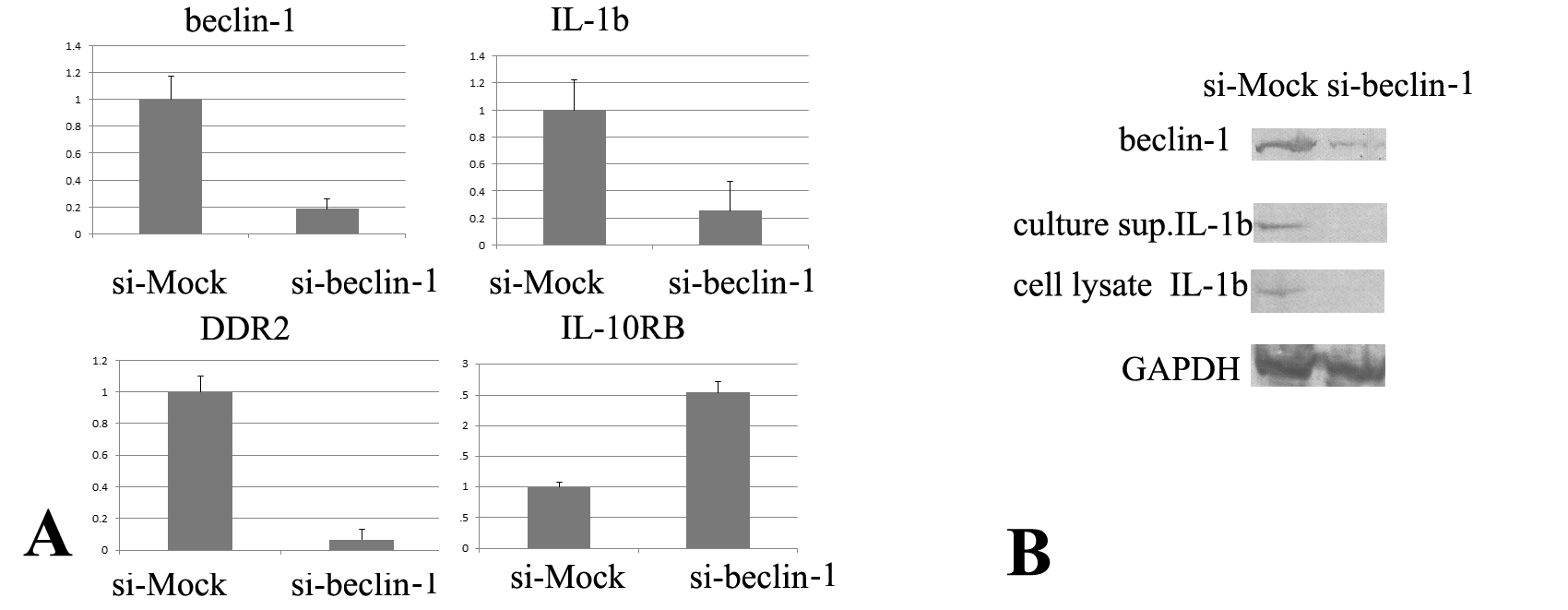

Supplement: S1 Fig — (TIF) [file pone.0125762.s001.tif]

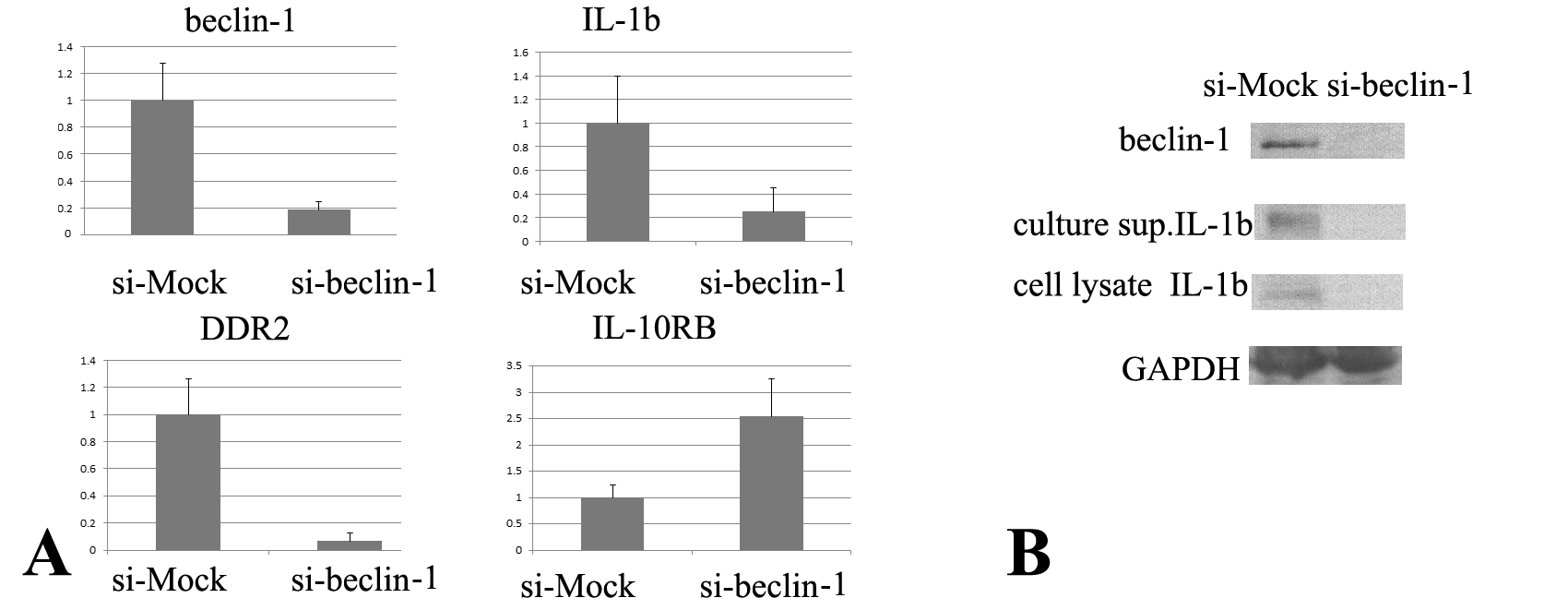

Supplement: S2 Fig — (TIF) [file pone.0125762.s002.tif]

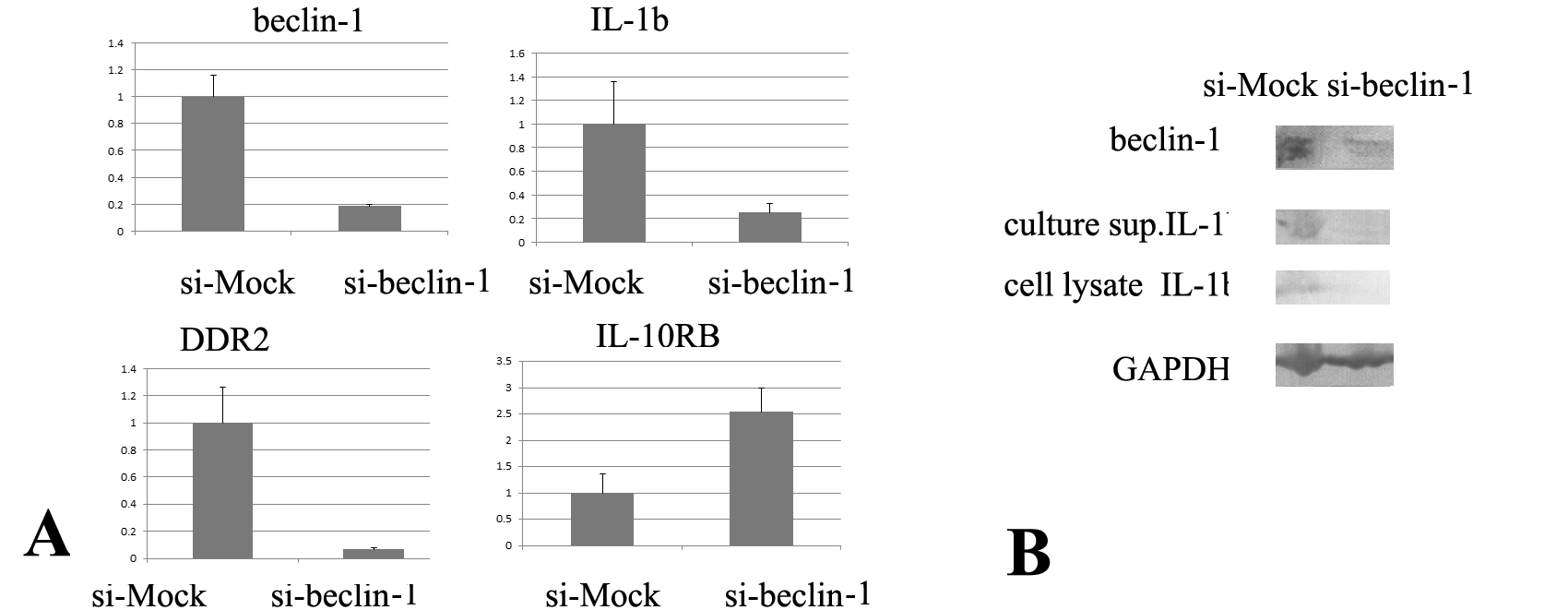

Supplement: S3 Fig — (TIF) [file pone.0125762.s003.tif]

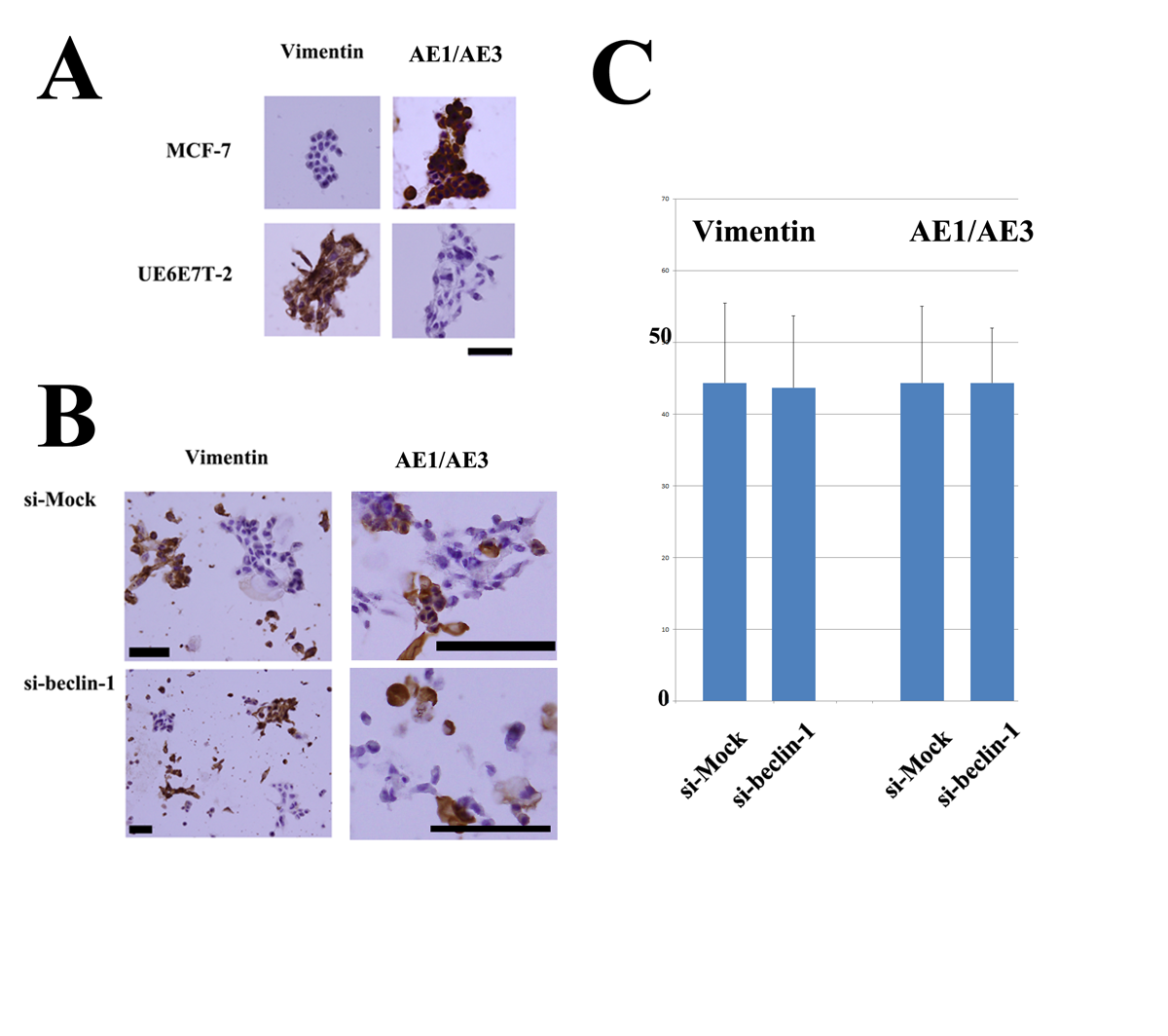

Supplement: S4 Fig — By contrast, UE6E7T-2 cells were stained with anti-vimentin antibody but not with AE1/AE3 antibody (A). At 48h after co-culture, the cells were collected by centrifugation, resuspended in 0.1ml of PBS, dropped on the slide glass, and dried at room temperature. After methanol fixation, the cells were immunostained with antibodies using the ImmPRESS polymerized reporter enzyme staining system (Vector laboratories). We calculated the stained cell numbers in representative x200 power field. We did not observe significant differences in the proportion of AE1/AE3- or vimentin-positive cells between the si-GFP- and si-beclin-1-treated groups. Representative vimentin and AE1/AE3 staining is shown in Figure (B), and the mean and SD are also shown (C). The experiments were performed in triplicate and significance was evaluated using the Student’s t-test. Scales bars: 100μm (TIF) [file pone.0125762.s004.tif]
